# Automated detection of Alzheimer’s disease: a multi-modal approach with 3D MRI and amyloid PET

**DOI:** 10.1038/s41598-024-56001-9

**Published:** 2024-03-03

**Authors:** Giovanna Castellano, Andrea Esposito, Eufemia Lella, Graziano Montanaro, Gennaro Vessio

**Affiliations:** 1 Department of Computer Science, University of Bari Aldo Moro, Bari, Italy; 2 Sirio - Research & Innovation, Sidea Group, Bari, Italy; 3 Tuidi s.r.l., Putignano, Italy

**Keywords:** Diagnostic markers, Alzheimer's disease

## Abstract

Recent advances in deep learning and imaging technologies have revolutionized automated medical image analysis, especially in diagnosing Alzheimer’s disease through neuroimaging. Despite the availability of various imaging modalities for the same patient, the development of multi-modal models leveraging these modalities remains underexplored. This paper addresses this gap by proposing and evaluating classification models using 2D and 3D MRI images and amyloid PET scans in uni-modal and multi-modal frameworks. Our findings demonstrate that models using volumetric data learn more effective representations than those using only 2D images. Furthermore, integrating multiple modalities enhances model performance over single-modality approaches significantly. We achieved state-of-the-art performance on the OASIS-3 cohort. Additionally, explainability analyses with Grad-CAM indicate that our model focuses on crucial AD-related regions for its predictions, underscoring its potential to aid in understanding the disease’s causes.

## Introduction

Dementia stands as a foremost challenge in global health, being a principal cause of disability and dependency among the elderly^1^. It affects approximately 55 million individuals worldwide, with a majority residing in low- and middle-income countries. This prevalence is set to rise alongside the increasing proportion of older individuals across the globe. Among the various forms of dementia, Alzheimer’s disease (AD) emerges as the predominant type, accounting for 60–70% of cases. While AD leads in prevalence, other significant forms such as vascular dementia, Lewy body dementia, and frontotemporal dementia also contribute to the overall burden of neurodegenerative disorders.

Despite the staggering prevalence, the quest for a cure for dementia, and AD in particular, remains elusive. Current pharmacological interventions offer limited efficacy, primarily catering to symptoms rather than underlying causes. Therefore, the importance of early diagnosis cannot be overstated, as it enables timely and optimal management strategies that significantly benefit patients, families, and caregivers alike.

In this context, the potential of Artificial Intelligence (AI) to transform dementia diagnosis, especially for AD, is increasingly recognized. AI offers a promising avenue to augment traditional diagnostic methods, leveraging advanced machine and deep learning techniques to harness reliable *biomarkers* for early and accurate detection.

In recent years, neuroimaging techniques, particularly Magnetic Resonance Imaging (MRI), have emerged as promising biomarkers in the preclinical stages of AD. MRI, utilizing magnetic fields and radio waves, generates high-quality two- or three-dimensional images of brain structures without requiring X-rays or radioactive tracers. This technology has significantly contributed to the development of diagnostic models for AD, offering a non-invasive method to detect patterns of brain atrophy indicative of the disease^2–4^.

Moreover, advancements in amyloid Positron Emission Tomography (PET) imaging have provided additional insights into the pathophysiology of AD^5^. Amyloid PET scans, by revealing amyloid plaques in the brain—previously identifiable only through autopsy—offer a crucial biomarker for evaluating cognitive impairment^6^. These scans employ radiotracers to visualize brain activity, with PiB-PET, AV45-PET, and FDG-PET being the principal variants used in AD diagnosis^7^. Each type differs in the radiotracer used: PiB-PET utilizes Pittsburgh Compound B (PiB) for amyloid binding; AV45-PET employs florbetapir (AV45), similar to PiB, for amyloid; and FDG-PET, distinct from the former, assesses glucose metabolism in the brain. The classification of PET scans into amyloid and glucose PETs highlights their diagnostic utility, with amyloid PETs showing higher sensitivity for AD diagnosis^8^.

Recent advancements in machine learning, particularly deep learning, have opened new avenues for automatically diagnosing Alzheimer’s disease using neuroimaging techniques such as amyloid PET^9^. Studies have shown that deep learning can significantly enhance the development of computer-aided detection systems using PET scans, mirroring successes previously documented with MRI data. However, much of this research has focused on uni-modal methods that rely solely on a single imaging modality, either MRI or PET. Despite the potential benefits, multi-modal approaches that integrate data from both MRI and PET scans remain underexplored. These approaches offer a promising avenue for gaining more comprehensive insights into brain abnormalities and improving diagnostic accuracy.

This article addresses this gap by proposing and evaluating Convolutional Neural Network (CNN) models designed to automatically differentiate individuals with AD from healthy controls. Using the OASIS-3 dataset^10^, we explore the utility of both 2D and 3D MRI and PET scans in uni-modal and multi-modal configurations, diverging from the predominant focus on single-modality analyses in existing literature.

Furthermore, with the expanding role of machine learning and AI in various applications, the field of eXplainable Artificial Intelligence (XAI) has garnered significant interest^11^. The demand for transparency in AI decision-making is especially critical in medicine, where opaque algorithms’ ethical and safety implications cannot be understated^12^. Given the complexity of early AD diagnosis, we have incorporated a *post-hoc* explanation method^13^ to provide insights that may illuminate the underlying mechanisms of the disease.

The rest of this paper is structured as follows. “Related work” reviews the literature pertinent to our study. “Materials” and “Methods” detail the dataset, data processing techniques, and our adopted methodologies. “Experiments” outlines our experimental design and discusses the findings. “Conclusion” summarizes our contributions and suggests directions for future research.

## Related work

Recent advancements in deep learning have revolutionized the diagnosis and detection of diseases, including Alzheimer’s, by offering a significant advantage over traditional machine learning algorithms. This advantage primarily lies in deep learning’s capacity to automatically extract features from data, eliminating the complexity and potential bias associated with manual feature extraction. In neuroimaging analysis for AD diagnosis, a substantial focus has been leveraging MRI data.

Among noteworthy contributions, Altay et al.^14^ recently proposed two different attention-based models and compared their performance with a 3D CNN baseline. The first attention model is a recurrent attention network, which extracts glimpses from stacked MRI images and feeds them into recurrent attention units to obtain a classification. The second model is a modified and repurposed Transformer, which first extracts the features of an image sequence from a pre-trained network and then feeds these features to a Transformer network to classify the sequence. The Transformer model outperformed the other approaches by achieving $\sim 91\%$ accuracy in preclinical AD detection on OASIS-3 data. Even more recently, Helaly et al.^15^ proposed two methods to classify MRI images and detect AD. The first method uses simple CNN architectures that deal with 2D and 3D structural brain scans from the ADNI dataset based on 2D and 3D convolutions. The second method applies the transfer learning principle to exploit pre-trained models for medical image classification. The latter strategy achieved a high accuracy of approximately 97% for multi-class AD stage classification.

While structural MRI has been the cornerstone of AD research, recent studies have also highlighted the significant role of amyloid PET in enhancing diagnostic capabilities. Amyloid PET offers a complementary perspective by enabling the detection and quantification of $\beta$-amyloid deposits in the cerebral cortex, a hallmark of AD. This technique uses contrast materials to visualize and measure the presence of $\beta$-amyloid and other substances, providing a more comprehensive understanding of the disease’s neuropathology.

In the pursuit of automated diagnosis, De Vries et al.^16^ demonstrated the utility of amyloid PET by training a 2D CNN model on sagittal FDG-PET images from the SCIENCe cohort and ADNI. Their model achieved a remarkable 95% accuracy in classifying A$\beta$ positive and negative scans among patients with cognitive decline. Reith et al.^17^ also pursued a similar goal. However, neither study specifically focused on distinguishing between cognitively impaired patients and healthy controls.

Addressing this gap, Tufail et al.^18^ investigated the effect of data augmentation techniques on CNN performance for early AD diagnosis using 3D PET scans from the ADNI dataset. Their research found that while the best-reported accuracy reached approximately 86%, combining all augmentation techniques did not yield optimal results. Our preliminary work achieved an 83% accuracy rate using 3D amyloid PET scans from the OASIS-3 dataset^19^. Although this did not represent a state-of-the-art outcome, it provided a promising basis for exploring multi-modal diagnostic strategies.

The exploration of multi-modal neuroimaging for the automatic detection of AD remains relatively underexplored, with few studies aiming to maximize performance by leveraging the combined strengths of different imaging modalities. Zhou et al.^20^ introduced a novel three-stage deep feature learning and fusion framework, achieving an impressive 89% accuracy in differentiating healthy individuals from those with AD using combined MRI and PET data from the ADNI dataset. However, this study did not explore the potential benefits of more sophisticated convolutional or volumetric approaches.

Lu et al.^21^ advanced the field with a multi-modal and multi-scale deep learning framework utilizing MRI and FDG-PET data, reaching an accuracy of approximately 85%. Despite its promise, this method’s computational intensity and disregard for the intrinsic three-dimensionality of neuroimaging data, due to its reliance on segmented image patches, present significant limitations. Liu et al.^22^ explored a cascade framework that employs 3D CNNs for feature extraction from local image patches, followed by a feature ensemble through a 2D CNN, culminating in a final classification layer. This approach demonstrated a notable accuracy of around 93% on the ADNI dataset.

Huang et al.’s work^23^, closely related to our research, employed a VGG-inspired 3D CNN to process MRI and FDG-PET data simultaneously, yielding a remarkable 90% accuracy in distinguishing between healthy subjects and those with AD. Song et al.^24^ adopted a distinct strategy by creating a synthetic “fused” volume from MRI and PET modalities rather than extracting and combining features separately.

Qiu et al.^25^ developed deep learning models to classify cognitive status using MRI, non-imaging factors, and their combinations, employing SHapley Additive exPlanations (SHAP) to link model predictions with established anatomical and pathological markers of neurodegeneration. Their findings support the potential of deep learning-driven techniques to match clinical diagnostic standards across varied datasets. Kong et al.^26^ introduced an image fusion method that integrates MRI and PET images from AD patients, using 3D CNNs for feature extraction to harness enriched multi-modal information. This is then analyzed by a fully connected neural network for classification and prediction. Rallambandi and Seetharaman^27^ proposed a deep learning-based Inception-ResNet50 wrapper model for distinguishing Mild Cognitive Impairment (MCI) and AD dementia patients from healthy controls, leveraging both structural MRI for spatial detail and functional PET for temporal resolution, underscoring the value of combining multi-modal imaging modalities.

Furthermore, a study by Gravina et al.^28^ proposed a Multi Input-Multi Output 3D CNN for assessing dementia severity, exploiting MRI and PET scans. This model adapts training iterations based on input characteristics and employs a joint fusion approach to manage incomplete acquisitions, effectively handling scenarios where one modality is missing. The architecture uses separate CNN networks for MRI and PET when both modalities are available, combining their outputs for the final classification. This approach ensures flexibility in handling data variability and completeness.

Adarsh et al.^29^ combined CNNs with multi-feature kernel supervised within-class-similar discriminative dictionary learning (MKSCDDL) to create an integrated diagnostic framework. This innovative model classifies individuals into AD, MCI, and cognitively normal categories, further discerning subtle phases within the MCI spectrum. It provides personalized evaluations and accentuates specific neuroanatomical regions linked to cognitive decline, illustrating the depth of insights achievable through advanced multi-modal analysis.

These findings underscore the potential of multi-modal neuroimaging in enhancing the accuracy of AD diagnosis, motivating our investigation into this promising avenue using the recently released OASIS-3 dataset. The consistent outperformance of multi-modal models over their uni-modal counterparts in existing studies highlights the importance of further exploration in this direction.

## Materials

In this study, we employed the OASIS-3 dataset^10^, the most recent iteration of the OASIS series, increasingly recognized as a benchmark for diverse research goals within the scientific community^30–33^. The dataset is publicly available (https://www.oasis-brains.org), ensuring broad accessibility for research purposes, with data sharing facilitated by participant consent. It encompasses MRI and PET images from 1098 participants, ages 42 to 95, and includes cognitively normal adults (605) and individuals at various stages of cognitive decline (493).

Our analysis specifically focused on amyloid PET and T1-weighted MRI scans. When feasible, the data collection protocol involved participants undergoing simultaneous PET and MRI scans within a 70-min session, beginning at the tracer injection time, to ensure synchronized acquisition of dynamic PET data alongside standard MRI. In instances where simultaneous imaging was not possible, PET and MRI scans were conducted in separate sessions, typically within a six-month interval, to maintain the relevance of the data across both modalities^10^.

From the 1607 PET sessions in the dataset, we selected 1352 sessions that had been processed using the PET Unified Pipeline, standardizing the spatial resolution to 8mm to reduce variability across scanners. The OASIS-3 dataset includes various types of PET scans, namely PiB-PET, AV45-PET, and FDG-PET, with varying availability by session. For our purposes, we exclusively analyzed amyloid PET scans (PiB-PET and AV45-PET), representing about 93% of the PET scan subset.

To enhance the accuracy of patient labeling and minimize the incidence of false negatives and false positives within our dataset, we employed a targeted algorithm for label correction. This approach was designed to identify and adjust labels based on the temporal sequence of patient diagnoses, ensuring greater consistency and reliability in our data. The algorithm operates under the following criteria:

- For a patient initially labeled as negative, if at least one of the two preceding labels (calculated by days since initial entry) was positive, and at least one of the following two labels was also positive, the patient’s label was adjusted to positive.
- Conversely, for a patient initially labeled as positive, if at least one of the two labels immediately before was negative, and at least one of the following two labels was negative, the patient’s label was revised to negative.

**Figure 1:**
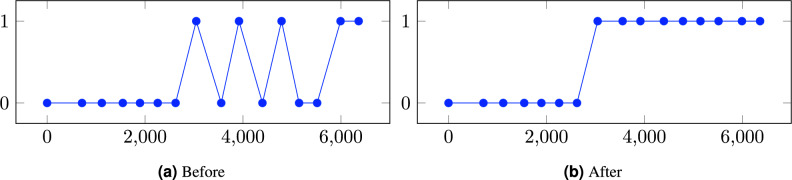
This figure illustrates the adjustment in label distribution for subject “OAS30040” before and after the post-processing step designed to mitigate false positives and false negatives. The horizontal axis represents the timeline of the subject’s participation in the study, measured in days since their initial entry.

Figure 1 illustrates this post-processing approach, showing how labels were adjusted to more accurately reflect the progression of disease status over time based on the sequence and consistency of diagnostic labels.

Our analysis labeled each PET scan according to the most recent diagnosis available. Given that not every scan session was directly accompanied by a psychiatric or neurological evaluation, we linked each scan with the temporally closest diagnostic test, regardless of whether it occurred before or after the scan. This process yielded a dataset comprising 1217 scans labeled as negative and 135 as positive. Among the positive scans, 20 were from subsequent visits of patients who maintained a positive diagnosis. Excluding these repetitive positive scans reduced the total count of unique positive cases to 115. We employed a two-level approach to address the significant imbalance between the negative and positive classes: random under-sampling of the negative class and data augmentation techniques applied to the positive class. The under-sampling process decreased the number of negative scans to 148, enhancing class balance. Further balancing was achieved by applying a series of rotation and mirroring operations to randomly selected positive scans, a method inspired by recent studies on this dataset^14^.

The MRI component of our study mirrored the PET scan analysis. Each MRI was paired with the corresponding PET Unified Pipeline scan, ensuring consistency across modalities. However, when multiple T1-weighted MRI scans were available for a single session, we selected only the most recent scan for inclusion in our dataset.

## Methods

### Classification models

Our models leverage a Convolutional Neural Network as the foundational architecture, tailored to accommodate the specific characteristics of the input scans—either in terms of their dimensionality or modality. The core architecture consists of four convolutional layers, each succeeded by a max pooling layer and batch normalization, cumulatively forming the feature extraction block of the network. The convolutional layers progressively increase in filter count, enhancing the network’s ability to capture complex features. Following the feature extraction phase, the architecture transitions into the classification block, which is composed of fully connected layers. This block processes the flattened output from the preceding block, incorporating a dropout layer (set at a 30% rate) to mitigate overfitting. The final layer, equipped with a sigmoid activation function, outputs a probability indicating the likelihood of the positive class, with values ranging from 0 to 1.

The rationale behind selecting CNNs as the foundation of our models is rooted in their proven capability and efficiency in handling image data, particularly in medical imaging and diagnostics. CNNs excel in automatically detecting critical features without the need for manual extraction, making them ideal for analyzing complex neuroimaging data.

Preparation of the data for training necessitated several preprocessing steps. Given the 4D nature of PET scans (3D images acquired over time), we condensed the volumetric data into a single “average” image, thereby simplifying it to a 3D format comparable to MRI scans. Subsequent steps involved isolating the brain within the scans. This was achieved by applying a Gaussian blur (kernel size of $13 \times 13$ and $\sigma = 150$) to the images to enhance segmentation via Otsu’s threshold method, followed by bounding box identification based on the images’ high-intensity regions. To standardize the input data, both PET and MRI images were resized to $128 \times 128$ voxels, the smallest dimension present in the dataset, to prevent upscaling artifacts. Owing to computational limits, incorporating the entire 3D volume for training was impractical. Thus, we selectively used the central 50 slices of each scan, ensuring the inclusion of significant brain regions without necessitating excessive computational resources. In models designed for 2D input, these slices were treated as independent images.

Our study systematically explored several variations of the proposed CNN architecture, tailored to different imaging modalities and dimensionalities, as outlined below:

- *2D MRI*: This model processes 2D MRI slices, employing 2D convolutional and pooling layers for feature extraction.
- *2D PET*: Similar to the 2D MRI variant, this model is designed for 2D PET slices, using 2D convolutional and pooling layers.
- *3D MRI*: Adapted for 3D MRI volumes, this variant incorporates 3D convolutional and pooling layers to capture volumetric features.
- *3D PET*: This model is analogous to the 3D MRI variant but is specifically optimized for 3D PET scan volumes.
- *3D PET* $\rightarrow$ *MRI*: Employing transfer learning, this model starts with weights pre-trained on 3D PET scans and is fine-tuned on 3D MRI volumes. This approach leverages learned features from PET scans to enhance MRI scan analysis, freezing the feature extraction layers and retraining only the classification block.
- *3D MRI* $\rightarrow$ *PET*: Mirroring the previous model but with reversed modality training—initially trained on 3D MRI and fine-tuned on 3D PET.
- *3D MRI + PET* (input): A single-branch, multi-modal model trained alternately on both 3D MRI and PET scans. This versatile model can predict based on whichever scan type is available, making it practical for varied clinical scenarios.
- *3D MRI + PET* (fusion): A dual-branch, multi-modal model that processes MRI and PET scans in parallel, combining features before classification. Each branch functions as a “twin” network, allowing for the distinct extraction and fusion of features from both modalities. Unlike the input model, this fusion approach requires both MRI and PET scans for prediction, offering a comprehensive but less flexible solution.

**Figure 2:**
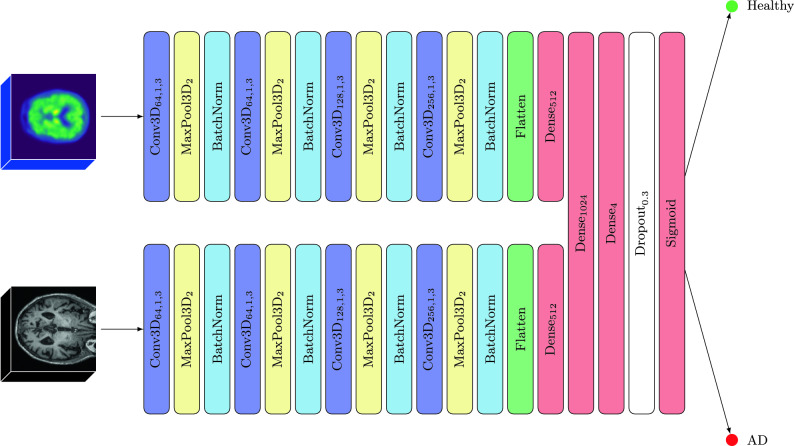
Architecture of the fusion model.

For all models, binary cross-entropy was used as the loss function, defined as in Eq. (1):

1 $$\begin{aligned} {\mathscr {H}}(y, {\hat{y}}) = -\left( y \log ({\hat{y}}) + (1 - y) \log (1 - {\hat{y}}) \right) , \end{aligned}$$

where *y* represents the true label, and ${\hat{y}}$ is the predicted probability generated by the model. Figure 2 shows the architecture of the fusion model; the others mirror this architecture with the appropriate modifications to make them work in a single mode or with 2D data.

### Explanation of the classification outcomes

Our models, inherently “black-box” in nature, do not offer interpretable or explainable outcomes without further analysis. To bridge this gap, we applied Gradient-weighted Class Activation Mapping (Grad-CAM), a technique proposed by Selvaraju et al.^13^, designed to elucidate the decision-making process of CNNs. Grad-CAM generates coarse localization maps that visually emphasize the regions within the original image most influential in predicting a specific concept.

The essence of Grad-CAM lies in its ability to create class-discriminative localization maps, $L^c_{GradCAM} \in {\mathbb {R}}^{u \times v}$, by leveraging the gradients of the class score, ${\hat{y}}^c$, for any given class *c*, against the convolutional layer’s feature maps, $A^k$. This process involves global average pooling of the gradients to ascertain the neuron importance weights, $\alpha _k^c$, which serve as a condensed representation of how each feature map contributes to the prediction of the target class. The computation of these weights is encapsulated by Eq. (2):

2 $$\begin{aligned} \alpha ^c_k = \overbrace{\frac{1}{Z}\sum _{i}\sum _{j}}^\text {global average pooling} \underbrace{\frac{\partial {\hat{y}}^c}{\partial A^k_{ij}}}_\text {gradients via backprop} \end{aligned}$$

In the formula, *i* and *j* refer to the location of the (*i*, *j*)-th pixel, while *Z* represents the total pixel count. A weighted combination of forwarding activation maps is then applied to obtain a coarse heatmap having the same size as the chosen convolutional layer. ReLU is then applied to obtain those features that have a positive influence on the class of interest, resulting in Eq. (3):

3 $$\begin{aligned} L^c_{GradCAM} = ReLU \underbrace{\left( \sum _{k}\alpha ^c_k A^k\right) }_\text {linear combination} \end{aligned}$$

By overlaying the Grad-CAM heatmaps onto the AAL2 atlas—a comprehensive anatomical atlas featuring 120 cortical and sub-cortical brain regions^34^—we were able to pinpoint the brain areas most significantly implicated in the model’s classifications. Regions with mean Grad-CAM values in the top 90th percentile were identified as critical for the model’s decision-making process, highlighting their potential relevance to AD pathology.

## Experiments

### Experimental setting

Our experimental framework was built on Google Colab, using an NVIDIA Tesla P100 GPU to ensure efficient computation. The architecture of our models was developed with the TensorFlow library, a choice inspired by its widespread acceptance and robust capabilities. The source code for our implementation is openly accessible (https://github.com/montanarograziano/Multimodal-approach-for-AD). The dataset underwent a patient-wise random split, allocating 80% to the training set and the remaining 20% to the test set. To fine-tune the hyperparameters, we employed an internal 10-fold *stratified* cross-validation approach. This technique ensures each fold maintains a balanced representation of class labels, mirroring the overall dataset distribution.

Optimization across all models was achieved through the Adam optimizer, with a dynamic learning rate and an exponential decay of 0.96. We initiated the learning rate at $5 \times 10^{-5}$. The training process was designed to run for up to 10,000 epochs, incorporating an early stopping mechanism activated after 35 epochs without improvement in validation accuracy. This criterion ensured the retention of the optimal model weights that yielded the highest validation accuracy.

Evaluation of model performance was conducted using established diagnostic metrics:

- *Accuracy*: Measures the overall proportion of true positive and true negative predictions across the dataset, providing a straightforward indicator of model performance.
- *Sensitivity*: Also known as the true positive rate, this metric quantifies the model’s ability to identify positive cases correctly. High sensitivity implies few false negatives, making it critical for conditions where missing a positive case has severe implications.
- *Specificity*: Reflects the model’s proficiency in identifying negative cases accurately, with high specificity indicating minimal false positives. This is crucial in avoiding unnecessary concern or treatment for healthy individuals.
- *AUC*: This represents the model’s capability to distinguish between classes across varying thresholds. A higher AUC value signifies better overall performance, encapsulating the trade-off between sensitivity and specificity.

### Classification results

Our investigation observed several critical insights from the classification results obtained using various model configurations on the OASIS-3 dataset (Table 1). Notably, models leveraging three-dimensional inputs outperformed their two-dimensional counterparts, likely due to the additional spatial information in 3D scans that facilitates learning more complex features. Furthermore, our analysis revealed that MRI scans in 2D or 3D formats consistently provided superior results to amyloid PET scans, with an accuracy difference of approximately 8-10%. While PET scans alone show promise, our findings reinforce that MRI scans are inherently more informative for our study. Interestingly, transfer learning between modalities did not yield improvements over uni-modal or multi-modal approaches. This suggests that features specific to one scan type might not directly apply to another, underscoring the complexity of cross-modality feature applicability. Excluding the robust performance of the 3D MRI model, multi-modal strategies, especially the fusion model, exceed uni-modal and transfer learning approaches. With the fusion model reaching an impressive 95% accuracy, our analysis validates the superiority of integrating multiple neuroimaging modalities. This suggests that MRI and PET scans fulfill complementary roles in disease prediction, with MRI proving crucial in uni-modal scenarios. The fusion model’s remarkable sensitivity is particularly advantageous for disease detection, where minimizing false negatives is critical, even though the 3D MRI model showed higher specificity. Given the priority of detecting disease presence accurately, the fusion model’s high sensitivity is deemed more beneficial for clinical applications, as it ensures that fewer cases are overlooked at the initial screening stage. Notably, the combined input model, which leverages both PET and MRI data, offers a balanced solution, bridging the gap between PET-specific and MRI-specific models with accuracies of approximately 81 and 91%, respectively. This model’s versatility in handling PET or MRI data makes it valuable, particularly in diverse clinical settings where scan availability may vary. Moreover, the prioritization of sensitivity in our evaluation reflects a strategic choice to favor detecting as many true positive cases as possible, recognizing the critical nature of early disease identification and intervention.

In analyzing the performance of our best-performing model, the fusion approach, it is noteworthy that all false negatives were from individuals with a clinical dementia rating score of 0.5. This score places these patients in a “gray zone”, where the detection of AD presents a more significant challenge due to the subtlety of the symptoms and the early stage of cognitive decline. Despite this inherent difficulty, it is remarkable that our model was able to correctly classify 80% of all patients within this ambiguous category in the test set. This high rate of accurate classification underscores the fusion model’s effectiveness, particularly in steering the complexities of early-stage AD diagnosis, where traditional methods may fail.

In our literature review, as summarized in the “Related Work” section, our findings demonstrate a performance that aligns well with existing research utilizing the OASIS and ADNI datasets for AD detection through deep learning techniques. Notably, our approach consistently matches or surpasses the results of significant recent studies. Our results’ alignment with state-of-the-art performances further validates the multi-modal strategy’s value in enhancing AD diagnostic processes, affirming the importance of incorporating diverse neuroimaging data for more accurate classification.

**Table 1:** The classification performance of the different models, as evaluated on the test set, is presented in ascending order based on accuracy. All metrics are expressed as percentages.

Model	Accuracy	Sensitivity	Specificity	AUC
*3D MRI* $\rightarrow$ *PET*	70.00	80.00	60.00	72.00
*2D PET*	72.00	64.27	79.73	74.00
*2D MRI*	80.78	77.93	81.93	85.00
*3D PET*	81.67	76.27	78.79	81.00
*3D PET* $\rightarrow$ *MRI*	83.33	80.00	86.66	84.00
*3D MRI + PET* (input)	85.00	76.67	93.33	85.00
*3D MRI*	91.67	83.33	100.0	94.00
*3D MRI + PET* (fusion)	95.00	93.33	96.66	93.00

### Explainability results

Our study used Grad-CAM to identify the brain regions most instrumental for classification by our models, analyzing both MRI and PET scans across positive and negative AD groups. The findings in Table 2 reveal significant insights into the neuroanatomical basis of AD detection.

**Table 2:** Key brain regions identified by MRI and PET for both positive and negative AD groups.

Positive MRI	Negative MRI	Positive PET	Negative PET
Postcentral_L	Postcentral_L	Postcentral_L	Postcentral_L
Precentral_L	Precentral_L	Precentral_L	Precentral_L
Temporal_Mid_L	Temporal_Mid_L	Temporal_Mid_L	Temporal_Mid_L
Precuneus_R	Temporal_Mid_R	Precuneus_R	Precuneus_R
Parietal_Inf_L	Postcentral_R	Precuneus_L	Temporal_Mid_R
Temporal_Mid_R	Temporal_Sup_L	Cingulate_Mid_R	Precuneus_L
Precentral_R	Temporal_Sup_R	Frontal_Mid_2_L	Cingulate_Mid_R
Postcentral_R	Precuneus_L	Frontal_Mid_2_R	Frontal_Mid_2_L
Temporal_Sup_L	Temporal_Inf_R	Frontal_Sup_2_R	Frontal_Mid_2_R
Temporal_Sup_R	Temporal_Inf_L	Frontal_Sup_2_L	Frontal_Sup_2_R
Precuneus_L	Cingulate_Mid_R	Cingulate_Mid_L	Frontal_Sup_2_L
Temporal_Inf_R	Cingulate_Mid_L	Frontal_Inf_Tri_L	Cingulate_Mid_L

For MRI scans, a notable consistency exists in the regions highlighted across both groups, with the Temporal Lobe emerging as the most critical area for classification. This observation aligns with established research indicating Temporal Lobe atrophy as a critical predictor of AD^35–37^ and other dementia forms^37,38^, particularly emphasizing the significance of the Medial Temporal Lobe^39–44^. Interestingly, the Middle Cingulate Gyrus and the Left Inferior Parietal Gyrus were identified as unique indicators for the negative and positive groups^45–47^. Additionally, the Precentral Gyrus and Precuneus, both associated with AD pathology, were identified as relevant, supporting their roles as early biomarkers of AD^48,49^.

PET scans revealed a substantial overlap in significant regions between the positive and negative groups, similar to MRI findings. Apart from the Middle Temporal Gyrus, Precentral Gyrus, and Precuneus, the Frontal Gyrus (encompassing the superior, middle, and inferior triangular parts) was markedly significant in AD detection^50–52^. This is consistent with literature documenting the Frontal Lobe’s involvement in AD through alterations and hypometabolism, underscoring its importance in early-stage AD and frontotemporal dementia^53–57^.

**Figure 3:**
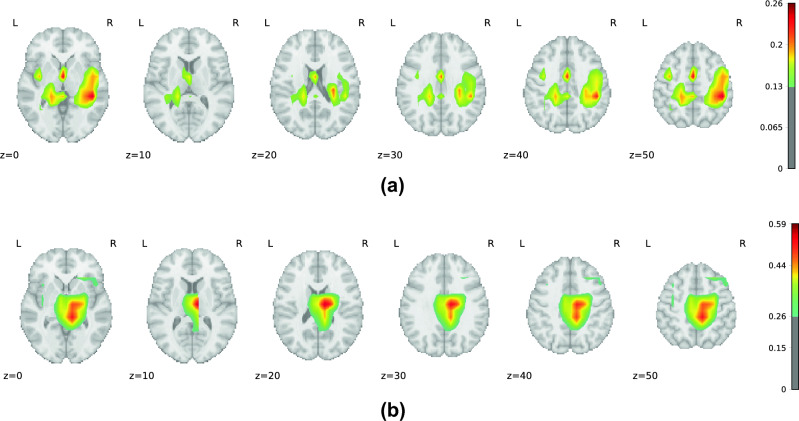
Three-dimensional visualizations created from multiple axial brain slices, showcasing Grad-CAM heatmaps for the positive class (**a**) MRI, (**b**) PET.

The overlap of significant regions across both groups, for MRI and PET, suggests that our model consistently focuses on the same areas for discrimination, albeit with some variation between modalities. This variation underscores the complementary nature of MRI and PET information, supporting our hypothesis that leveraging both modalities can enhance diagnostic accuracy. These insights are further illustrated in Fig. 3, showcasing the Grad-CAM heatmaps and the pivotal role of identified regions in AD prediction.

Overall, our findings substantiate the value of multi-modal imaging in AD detection, highlighting non-redundant, complementary information provided by MRI and PET scans. This approach aligns with existing neuropathological understanding and opens avenues for more subtle and effective diagnostic strategies.

## Conclusion

Our study explored the development of a multi-modal diagnostic model for AD, leveraging both 3D MRI and amyloid PET imaging. Our findings affirm the hypothesis that these modalities offer distinct yet complementary insights, enhancing the construction of predictive models for AD. The experimental outcomes, both quantitative and qualitative, demonstrate that our proposed approaches not only align with but also potentially surpass current state-of-the-art methods, identifying key brain regions associated with AD in concordance with contemporary research.

However, our study is not without its limitations. Due to computational constraints, we selected only 50 slices from the axial plane for both PET and MRI analyses, which, while practical, may not fully capture the comprehensive spatial information available across all three anatomical planes (sagittal, coronal, and axial). This selection process potentially limits the depth of our predictions and interpretations. Furthermore, our method of averaging frames over time, though necessary for the manageability of PET scans, results in a loss of temporal resolution that could otherwise offer additional diagnostic insights. Future research could benefit from integrating full volumetric (4D) data to preserve temporal dynamics despite the increased computational demands or employing models that pinpoint the most diagnostically relevant time frames.

Furthermore, our findings underscore that although PET scans exhibit promise, MRI scans are inherently more aligned with our study’s goals, providing richer diagnostic information. While our research did not directly target the classification of patients based on amyloid positivity or negativity, pursuing an in-depth analysis of amyloid PET’s capacity to differentiate amyloid-positive from -negative individuals promises to shed light on its utility, particularly for devising targeted interventions for neurodegenerative ailments.

Lastly, considering the pivotal involvement of the Hippocampus and Medial Temporal Lobe in AD pathology, comparing our models’ sensitivity and specificity with metrics derived from MRI measurements of these areas’ volumes could significantly enhance our insight into the comparative diagnostic value of diverse neuroimaging biomarkers. Our current focus has been on deploying Convolutional Neural Networks to analyze structural MRI and amyloid PET data, leveraging deep learning’s robust pattern recognition for a holistic evaluation of brain imaging data. Anticipating future research directions, integrating a comparative volumetric analysis represents a promising expansion of our work, aiming to combine the strengths of both volumetric and pattern recognition methodologies for a richer diagnostic toolkit.

## Data Availability

The OASIS-3 dataset is publicly accessible to the research community (https://www.oasis-brains.org). Interested users must review and agree to the OASIS data usage terms before gaining access to the dataset.
